# Recent Advances in the Role of Discoidin Domain Receptor Tyrosine Kinase 1 and Discoidin Domain Receptor Tyrosine Kinase 2 in Breast and Ovarian Cancer

**DOI:** 10.3389/fcell.2021.747314

**Published:** 2021-11-03

**Authors:** Li Chen, Xiangyi Kong, Yi Fang, Shishir Paunikar, Xiangyu Wang, James A. L. Brown, Emer Bourke, Xingrui Li, Jing Wang

**Affiliations:** ^1^Department of Thyroid and Breast Surgery, Tongji Hospital, Tongji Medical College, Huazhong University of Science and Technology, Wuhan, China; ^2^Department of Breast Surgical Oncology, National Cancer Center, National Clinical Research Center for Cancer, Cancer Hospital, Chinese Academy of Medical Sciences, Peking Union Medical College, Beijing, China; ^3^Discipline of Pathology, School of Medicine, Lambe Institute for Translational Research, National University of Ireland Galway, Galway, Ireland; ^4^Department of Biological Sciences, University of Limerick, Limerick, Ireland; ^5^Health Research Institute, University of Limerick, Limerick, Ireland

**Keywords:** discoidin domain receptor tyrosine kinases (DDR), receptor tyrosine kinase (RTK), protein tyrosine kinases (PTK), breast, ovarian, cancer, treatment, ECM

## Abstract

Discoidin domain receptor tyrosine kinases (DDRs) are a class of receptor tyrosine kinases (RTKs), and their dysregulation is associated with multiple diseases (including cancer, chronic inflammatory conditions, and fibrosis). The DDR family members (DDR1a-e and DDR2) are widely expressed, with predominant expression of DDR1 in epithelial cells and DDR2 in mesenchymal cells. Structurally, DDRs consist of three regions (an extracellular ligand binding domain, a transmembrane domain, and an intracellular region containing a kinase domain), with their kinase activity induced by receptor-specific ligand binding. Collagen binding to DDRs stimulates DDR phosphorylation activating kinase activity, signaling to MAPK, integrin, TGF-β, insulin receptor, and Notch signaling pathways. Abnormal DDR expression is detected in a range of solid tumors (including breast, ovarian, cervical liver, gastric, colorectal, lung, and brain). During tumorigenesis, abnormal activation of DDRs leads to invasion and metastasis, via dysregulation of cell adhesion, migration, proliferation, secretion of cytokines, and extracellular matrix remodeling. Differential expression or mutation of DDRs correlates with pathological classification, clinical characteristics, treatment response, and prognosis. Here, we discuss the discovery, structural characteristics, organizational distribution, and DDR-dependent signaling. Importantly, we highlight the key role of DDRs in the development and progression of breast and ovarian cancer.

## Introduction

Breast and ovarian cancer are amongst the most common female malignancies, with a history of breast cancer linked to a higher risk of ovarian cancer (Mahumud et al., 2019). Recent advances in medical science, including earlier detection and targeted treatments, have significantly increased survival in many cancers, including breast and ovarian (Matchett et al., 2017; Prakash et al., 2018; Siegel et al., 2020). The primary treatment of solid tumors is surgical excision combined with other therapeutic approaches, including systemic chemotherapy, radiation therapy and targeted therapies (including immunotherapies and drugs targeting disease specific mutations or proteins). However, further improvements in treatment efficacy and specificity are needed (Li et al., 2019; Momenimovahed et al., 2019). Targeted molecular therapy is a promising strategy utilized to impede cancer cell growth, invasion or metastasis by targeting the unique genetic, proteomic or epigenetic profile of individual tumors. The discovery, and understanding the mechanism of action, of novel target molecules dysregulated in female malignancies is central to the development of truly personalized cancer treatments needed to improve patient survival (Bax et al., 2016; Sapiezynski et al., 2016; Emens, 2018; Maennling et al., 2019).

Many novel therapeutics target extracellular molecules dysregulated in tumors (e.g., the Her2 receptor in breast cancer). This approach has the advantage of improved target access for drugs, with the therapeutics not requiring cell entry (Insua-Rodríguez and Oskarsson, 2016; Nakhjavani et al., 2019). Importantly, cell to extracellular matrix (ECM) contact (mediated by extracellular receptors) significantly regulates many aspects of tumor cell behavior, including proliferation, apoptosis, basement membrane invasion, and metastases (Marastoni et al., 2008). As a major component of tissue ECM, collagen exhibits disrupted architecture within the tumor microenvironment, and binding of collagen to tumor cells triggers wide-ranging causal effects on tumor development (Xu et al., 2019). Importantly, the direct binding of collagen to tumor cells is mediated by discoidin domain receptor tyrosine kinases (DDRs), a subfamily of the receptor tyrosine kinases (RTKs), which are promising new therapeutic molecular targets.

## Receptor Tyrosine Kinase Family: Structure and Characteristics

**FIGURE 1:**
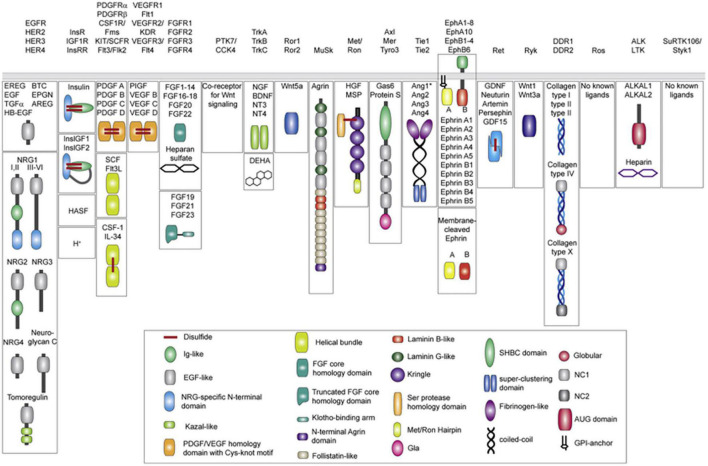
Receptor tyrosine kinase ligands (Human). A total of 19 distinct RTK families (including recent revision removing the now Ser/Thr receptor kinases classified LMR1–3 family). Ligands for each RTK family are shown underneath in mature, secreted form. All membrane-tethered ligands are cleaved off the membrane except for ephrins, which activate their cognate receptors in a juxtacrine fashion. The ligands are drawn with their N-terminus pointing away from the membrane. Main structural domains are depicted in a cartoon form in their known oligomeric state except for Angiotensins, which may form higher-order oligomers in addition to dimers. If applicable, domain labels are included as captions. Sizes of individual domains are not drawn to scale. Reprinted from Lemmon and Schlessinger (2010).

**FIGURE 2:**
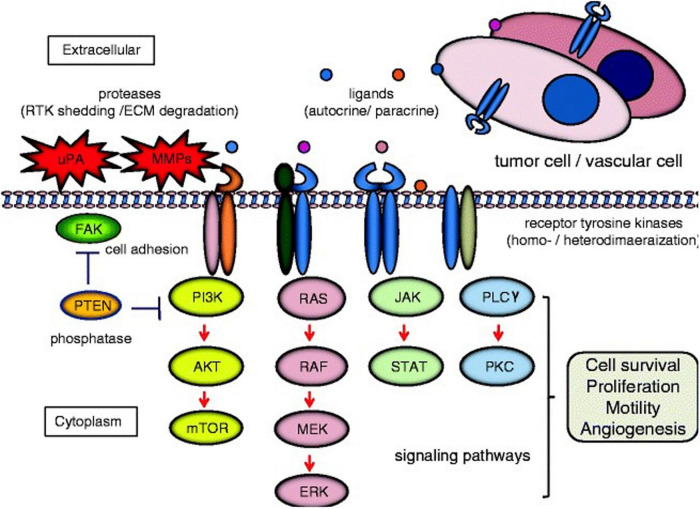
Cell signaling pathways induced by receptor tyrosine kinases (RTK). Homo-/heterodimerization of RTKs are caused by their ligands in autocrine or paracrine fashion. The dimerized receptors can initiate signal transduction cascades involved in cell survival, proliferation, motility, and angiogenesis e.g., phosphatidylinositol 3-kinase (PI3K)/AKT/mTOR; RAS/RAF/mitogen-activated protein kinase (MEK)/extracellular signal-regulated kinase (ERK); Janus kinase (JAK)/signal transducer and activator of transcription (STAT); and phospholipase Cγ (PLC)γ/protein kinase C (PKC). Examples of cross-talk between RTK signaling and proteins associated with cell invasion [e.g., urokinase-type plasminogen activator (uPA), matrix metalloproteinase (MMP)s, focal adhesion kinase (FAK), and phosphatase and tensin homolog deleted from chromosome 10 (PTEN)] are demonstrated. Red arrows and blue bars indicate activation and suppression, respectively extracellular matrix (ECM). Reprinted from Ghosh et al. (2020).

Protein tyrosine kinases (PTKs) are a class of protein kinases which require tyrosine phosphorylation for activation (Ruckert et al., 2019). Within the PTK superfamily, the RTK family are single transmembrane proteins (with over 20 classes identified), acting as both receptors and enzymes (Figure 1; Lemmon and Schlessinger, 2010). PTKs function as signal transducers, and as critical regulatory factors in many signaling pathways, affecting cell cycle, cell migration, metabolism, survival, and differentiation. RTKs include the DDR family, in addition to insulin receptors, epidermal growth factor receptors (EGFRs), platelet growth factor receptors (PGFRs), fibroblast growth factor receptors (FGFRs), vascular endothelial growth factor receptors, ephrin receptors, hepatocyte growth factor receptor, nerve growth factor receptors, and colon carcinoma kinase receptors (Lemmon and Schlessinger, 2010; Du and Lovly, 2018). RTKs have the same structural layout: the extracellular domain (containing a ligand-binding site), the hydrophobic alpha helix region (membrane spanning), and the intra-cellular domain (containing the kinase domain) (Julien et al., 2013). In cancer, many RTKs have been shown to play critical roles in tumorigenesis, development, and metastasis (Figure 2; Yamaoka et al., 2018; Ghosh et al., 2020).

## Discoidin Domain Receptor Tyrosine Kinases

Within the RTKs the family of discoidin domain receptor tyrosine kinases (DDRs) are non-integrated collagen receptors, where the extracellular domain contains a discoidin-like domain (or discoidin domain receptor). In mammals, there are two subtypes of DDRs, DDR1 and DDR2, the only RTKs known to interact with structural components of the ECM and in which soluble growth factors do not activate them (Vogel et al., 2006; Itoh, 2018). DDR1 and DDR2 function as ECM signal transducers, binding to the ECM collagen, and initiating intra-cellular signaling (Henriet et al., 2018). DDRs and their signaling pathways are promising targets for the development of new clinical treatments, particularly in cancers with altered DDR expression or function (Gao et al., 2021).

### Discoidin Domain Receptor Tyrosine Kinase Subfamily Discovery

Discoidin domain receptor tyrosine kinase 1 and discoidin domain receptor tyrosine kinase 2 contain a discoidin homology (DS) domain in their extracellular regions. The DDR1 subfamily is composed of five splice-variant isoforms (DDR1a-e) initially discovered in a screen for tyrosine phosphorylation in breast cancer cells (Johnson et al., 1993; Takai et al., 2018). The DDR2 subfamily (consisting of DDR2 alone) shares highly conserved sequences with the DDR1 family. The DDRs were initially classed as orphan receptors, until the discovery that many collagens function as their ligands (Shrivastava et al., 1997; Vogel et al., 1997). DDR1 is activated by binding of wide-ranging types of collagen (including collagen I, IV, V, VI, and VIII), whereas DDR2 is exclusively activated by the fibrillar collagens (type I, III, and type X) (Leitinger and Kwan, 2006).

### Discoidin Domain Receptor Tyrosine Kinase Protein Structure

**FIGURE 3:**
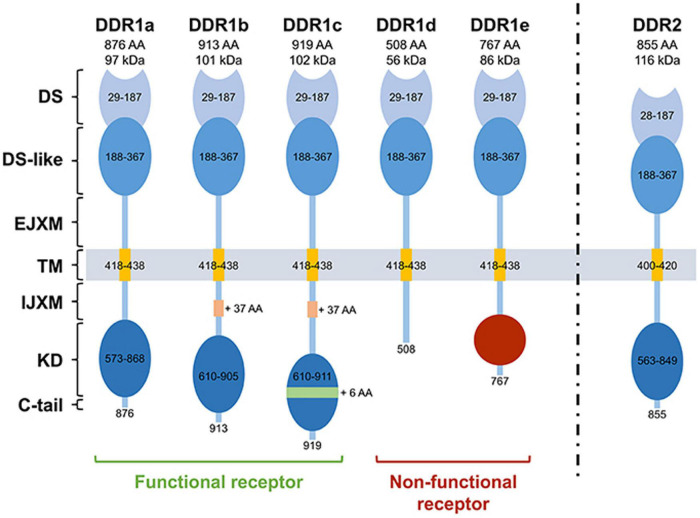
The structure of DDRs. DDR1a, DDR1b, DDR1c, and DDR2 are enzymatic active receptors, and DDR1d and DDR1e are inactive kinase-deficient receptors. DS, discoidin domain; DS-like, discoidin-like domain; EJXM, extracellular juxtamembrane region; TM, transmembrane segment; IJXM, intracellular juxtamembrane region; KD, kinase domain; AA, Amino Acid. Reprinted from Rammal et al. (2016).

The *DDR1* gene (6p21.33) contains 17 exons, which generates five DDR1 isoforms through alternative splicing. DDR1a-c are full-length functional receptors, while DDR1d and DDR1e are truncated receptors lacking kinase activity (Figure 3; Rammal et al., 2016). The canonical DDR1 protein contains 8 extracellular regions, 1 transmembrane region, 3 near membrane regions, and 5 tyrosine kinase catalytic regions (Moll et al., 2019). The best studied DDR1 isoforms are DDR1a and DDR1b. Notably, DDR1a is 876 amino acids (aa) long and has a 37 aa deletion in the transmembrane (TM) region and a 6 aa deletion in the kinase domain (KD) region (between exons 13 and 14) (compared to DDR1c). In contrast, DDR1b has an additional 37 aa in the intra-cellular juxtamembrane (IJXM) region. DDR1c is the longest isoform (919 aa), containing both the additional 37 aa in the IJXM, and an additional 6 aa in the kinase domain region. DDR1d is 508 aa long and lacks exons 11 and 12 causing a frameshift mutation that generates a stop codon and loss of the KD. DDR1e is 767 aa long and lacks exons 11 and 12 and the first half of exon 10, generating an inactive KD. In addition, DDR1d and DDR1e are inactive due to a lack of ATP binding sites, and currently have not had a clear biological function assigned to them (Valiathan et al., 2012). DDR1 can be cleaved (by an as yet undetermined protease) into a 54 kb soluble alpha subunit located in the extracellular region, and a 63 kb beta subunit anchored to the membrane (Yeh et al., 2009). DDR1 is activated by recognizing the GVMGVO peptide motif in fibrillar collagens (I, II, III, and VIII), and non-fibrillar basement membrane collagen IV (Xu et al., 2012). The *DDR2* gene is located in human 1q23.3, contains 19 exons and encodes a single transcript, and one 855 aa protein (Kim et al., 2017). The DDR2 juxtamembrane (JM) domain is composed of extracellular JM region (approximately 30 aa) followed by large cytosolic JM regions of about 142 aa. The KD is capped by a short C-terminal peptide of about 6 aa.

## Cellular Expression and Distribution

**FIGURE 4:**
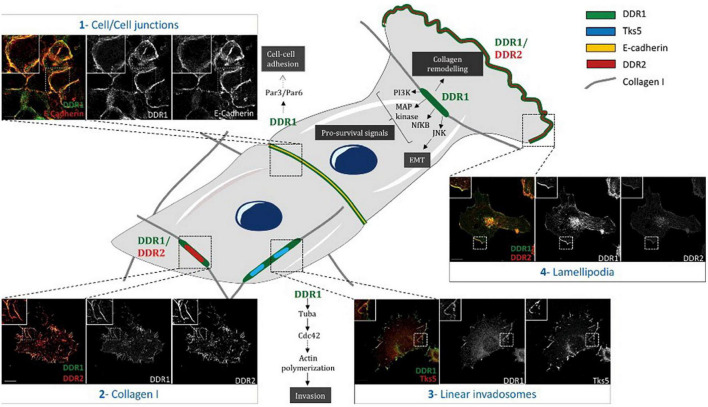
Differential subcellular localization of DDRs in cells. Schematic representation illustrating different subcellular localizations of DDRs in cells associated with their functions. (1) In A431 cells, DDR1 interacts with E- cadherin and the polarity complex Par3/Par6 in order to maintain cell/cell junctions. (2) In A375 cells, DDR1 and DDR2 colocalize together with fibrillar collagen type I. (3) In A375 melanoma cells, on a collagen I matrix, DDR1 co-localizes with Tks5 (a marker of invadosomes). DDR1 activation induces Tuba/Cdc42 pathway leading to linear invadosome formation. (4) In A375 melanoma migrating cells, both DDR1 and DDR2 co-localize with lamellipodia. Some pathways induced by DDR1 activation are represented in this schematic. Scale bar = 5 mM. Reprinted from Henriet et al. (2018).

While the DDR1 protein is primarily expressed in epithelial cells, expression is seen other cell types including the myelin sheath and microglia, keratinocytes, large intestinal epithelia, lung epithelia, breast epithelia, adrenal cortical cells, pancreatic ducts, and thyroid follicles (Barker et al., 1995). Physiologically DDR1 plays a key role in organogenesis, and in breast development (Moll et al., 2019). DDR2 is predominantly expressed in connective tissues of mesenchymal origin, with high expression also seen in the skeletal and heart muscle, as well as kidney and lung cells and immature dendritic cells (constitutive expression) (Alves et al., 1995; Vogel, 1999; Lee et al., 2007). According to the microenvironment, DDRs can form multiple subcellular complexes, with diverse functions (Figure 4; Iwai et al., 2014; Henriet et al., 2018; Agarwal et al., 2019).

## Discoidin Domain Receptor Tyrosine Kinase 1 and Discoidin Domain Receptor Tyrosine Kinase 2 Mediated Signaling Cascades

**FIGURE 5:**
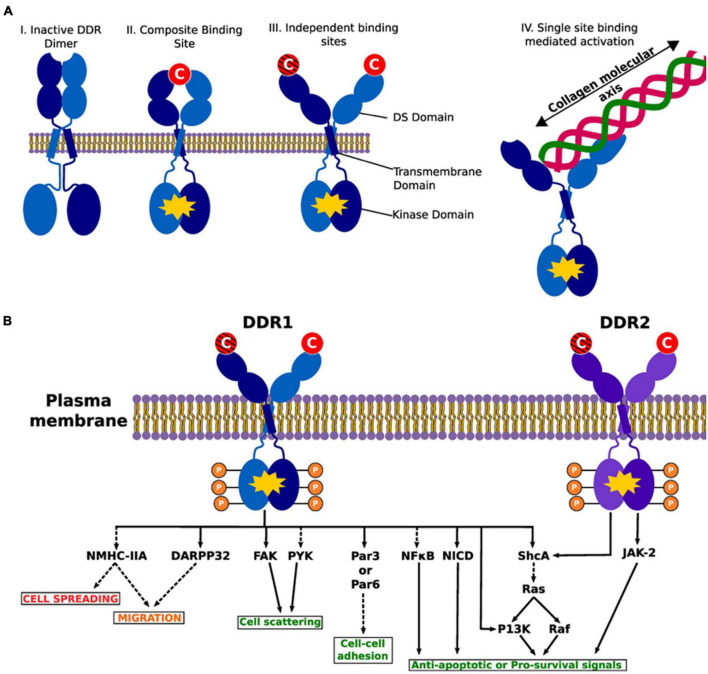
Illustration of DDR activation by fibrillar collagens. Activated dimers are depicted with a yellow polygon on the intracellular kinase part of the dimer. **(A)** The various possible conformations of the DDR1 dimer in active and inactive states. DDRs are activated upon binding with fibrillar collagens in the DS domain. (I) An inactive DDR dimer. (II) Activated DDR dimer by composite binding. Here the collagen binding sites from each monomer create a “composite” binding site to bind to collagen to activate the intracellular kinase. (III) Individual binding sites on each monomer may interact with different collagen monomers leading to activation. The crosshatched collagen monomer illustrates that binding to both domains at the same time may or may not be necessary for activation (i.e., it is possible that binding of a single collagen molecule to a single DS domain activates the entire complex). (IV) As alluded to in III, a single collagen molecule binds just one DS domain. Illustrates the collagen molecular and DDR interaction. We present previous evidence of a collagen engagement on the binding site of a monomer leads to DDR activation. II–IV represent possible means of activation. **(B)** A list of common intracellular targets and cellular cascades that result from DDR activation. Phosphates are shown to demonstrate the kinase activity of the DDRs. The red text shows processes that are suppressed by DDR signaling, green text represents processes that are promoted and the orange text represents processes that are either suppressed or promoted. Dashed lines indicate indirect activity and solid lines show direct interaction and effects. DARPP-32, dopamine- and cAMP-regulated phosphoprotein, Mr 32 kDa; FAK, focal adhesion kinase; JAK-2, janus kinase 2; NF-κB, nuclear factor kappa B; NICD, notch 1 intracellular domain; NMHC-II, non-muscle myosin IIA heavy chain; P13K phosphatidylinositol 3-kinase; Par3/Par6, cell polarity regulators; PYK, protein tyrosine kinsase; ShcA, SH2 containing transforming protein A. Reprinted from Orgel and Madhurapantula (2019).

**FIGURE 6:**
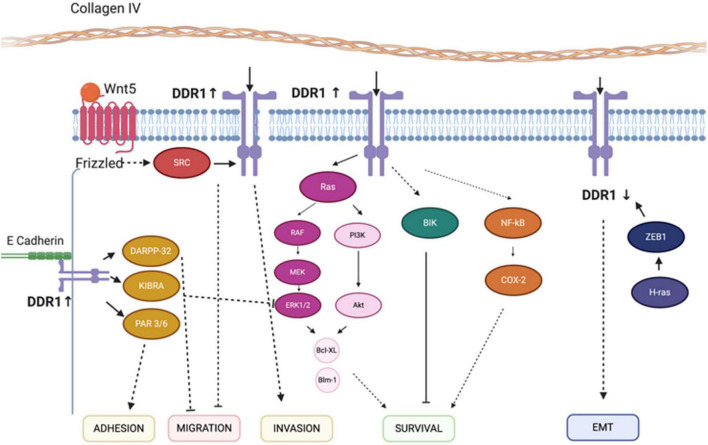
Discoidin domain receptor tyrosine kinase 1-associated signaling pathways. The mechanisms for the effect of ZEB1, COX-2, DARPP-32 and Wnt-5a on the migration, survival, EMT, and invasion regulatory networks are illustrated. Solid lines indicate direct interactions or effects, whereas, dashed lines indicate indirect interactions or effects through one or more intermediate steps. Pointed and flat arrows indicate activating and inhibiting effects, respectively. DDR1, discoidin domain receptor 1; ZEB1, zinc finger E-box-binding homeobox 1; COX-2, cyclooxygenase-2; DARPP-32, dopamine- and cAMP-regulated neuronal phosphoprotein; EMT, epithelial-to-mesenchymal transition; CD9, cluster of differentiation 9; NMHC-IIA, non-muscle myosin heavy chain-IIA; BIK, Bcl-interacting killer; NF-κB, nuclear factor-κB; MEK, ERK activator kinase; ERK, extracellular signal-regulated kinase. Figure adapted and modified from Payne and Huang (2014) and Jing et al. (2018).

**FIGURE 7:**
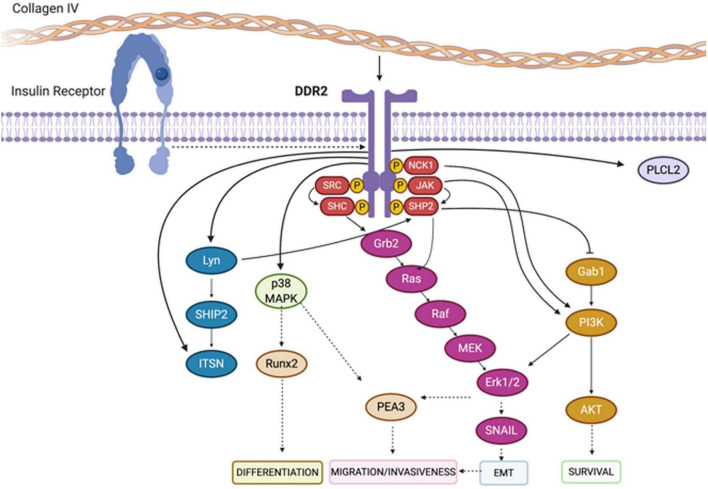
Depiction of signaling pathways activated downstream of DDR2. Binding of collagen to the extracellular domain of DDR2 triggers the auto-phosphorylation of its cytoplasmic domain. This results in the recruitment of downstream adaptor proteins, kinases and phosphatases including SHC, NCK1, SRC and SHP-2. As a consequence, a series of canonical signaling pathways are initiated including the Erk1/2 and PI3K cascades. Figure adapted and modified from Payne and Huang (2014) and Jing et al. (2018).

Discoidin domain receptor tyrosine kinase 1 and discoidin domain receptor tyrosine kinase 2 are non-integrin receptors acting as sensors of the ECM (activated by fibrillar collagens), mediating signaling which regulates many diverse cellular processes (including proliferation, invasion, migration, differentiation, cytokine secretion, ECM remodeling, and embryonic development) (Figure 5; Orgel and Madhurapantula, 2019; Vanajothi et al., 2019; Bonfil et al., 2021). Dysregulated DDR-mediated signaling is strongly associated with many forms of cancer, and has been associated with other diseases (including osteogenesis hypoplasia, and arthritis) (Carafoli and Hohenester, 2013). Overviews of key DDR1 or DDR2-mediated signaling cascades are shown in Figures 6, 7, respectively (Payne and Huang, 2014; Jing et al., 2018).

### Collagen-Mediated Discoidin Domain Receptor Tyrosine Kinase 1 and Discoidin Domain Receptor Tyrosine Kinase 2 Signaling

Collagen stimulation of DDR1 leads to binding and activation of Notch1 increasing cell survival, by promoting the activation of transcription factors (Hcs1 and Hcy2) and the expression of pro-survival genes (though γ-secretase cleavage of the Notch1 intracellular domain, and nuclear localization) (Chen L. Y. et al., 2019; Kim et al., 2019). Cell cycle regulated signaling mediates Wnt-5a expression, which can act as an upstream regulator of DDR1, promoting collagen-induced DDR1 phosphorylation and activation, which alters cell adhesion and migration (Dejmek et al., 2003, 2005). Collagen - has been demonstrated to trigger DDR1-induced Pyk2 phosphorylation, which induces apoptosis and inhibits epithelial to mesenchymal transition (EMT) (Huang et al., 2016). Furthermore, DDRs have been demonstrated to have a critical role in regulating keloid collagen overproduction, regulating collagen-fibroblast signaling (Jiang et al., 2009).

Additionally, DDRs are well known to differentially modulate distinctive mitogen-activated Protein Kinase (MAPK) signaling pathways, transducing extracellular signals and mediating cellular responses to these extracellular stimuli. In mammary epithelial cells and smooth muscle cells, DDR1 activates extracellular signal-regulated protein kinases (ERK)1/2 following extracellular stimulation (Peng et al., 2017). Conversely, in mesangial cells, DDR1 has been shown to inhibit ERK1/2 activation (Moll et al., 2018). DDR1 can mediate downstream signals via c-Jun N-terminal Kinases (JNK), as seen in pancreatic cancer cells (Zhu et al., 2019). Following DNA damage, p53 mediated DDR1 phosphorylation is involved in cell survival or apoptosis decisions, through Ras-Raf-MAPK and Protein kinase B (AKT) signaling (Ongusaha et al., 2003).

Overexpression of DDR1 promotes tumor cell proliferation (including regulating tumor-infiltrating CD4 + and CD8 + T cells), while silencing or knocking out DDR1 can reduce tumor cell growth (Peretti et al., 2019). Overexpression of DDR1/2 in cells expressing integrin α1β1 and α2β1 can enhance the level of integrin activation-mediated cell adhesion (Xu et al., 2012). While integrin β1 promotes cell differentiation by down-regulating E-cadherin expression, DDR1 promotes differentiation by increasing the stability of E-cadherin membrane proteins (Wang et al., 2005). Furthermore, loss of either E-cadherin or DDR1 is sufficient to promote increased cortical contractility, resulting in the loss of cell-cell adhesion (Rhys et al., 2018). It has been shown that DDR1 and integrin α2β1 can up-regulate N-cadherin by interacting with transforming growth factor β-inducing protein I (TGFβI), to promote the growth, invasion and metastasis (Krohn et al., 2016).

Interestingly, expression of DDR1 can be regulated by secretory pathway Ca2 + -ATPase (SPCA2) through collagen I and miR-199B-5p (Sun et al., 2018; Wu et al., 2018). Studies have found that in DDR1 knockout mice, collagen deposition is reduced, and the stromal-vascular fraction (SVF) of adipose tissue is impaired. SVF secretes the cytokine Interleukin-6 (IL-6) in a DDR1-dependent manner, and SVF produced IL-6 increases tumor cell invasion *in vitro* (Hansen et al., 2006).

Tumor growth requires invading cancer cells to acquire mechanisms to penetrate a highly reactive collagen-rich stroma which possess anti-proliferative and pro-apoptotic properties. DDR binding to collagen in the microenvironment can regulate both apoptosis and tissue remodeling, through modulating the expression and activity of matrix metalloproteinases (MMPs). Binding of stromal type I collagen to DDR1 on tumor cells, triggers a signaling pathway culminating in the transcriptional up-regulation of pro-apoptotic Bcl-2-interacting killer (BIK), promoting cell growth suppression and central mediator of chondrocyte markers type I collagen (COL1)-induced apoptosis (Maquoi et al., 2012; Saby et al., 2019). In addition, membrane-bound matrix metalloproteinase membrane-type-1 matrix metalloproteinase (MT1-MMP) is highly expressed in invasive cells, including fibroblasts and invasive cancer cells, and promotes breast cancer tumorigenesis by inhibiting the apoptosis induced by the collagen/DDR1/BIK signaling axis (Liu et al., 2014). MT1-MMP acts through the degradation of collagen fibers and/or cleavage of the DDR1 receptor (Assent et al., 2015). In addition, fibrillar collagen binding to DDR2 mediates MT1-MMP overexpression in fibroblasts (Majkowska et al., 2017). Knockdown of DDR2 inhibits collagen-induced MT1-MMP-dependent activation of pro-MMP-2, and the resultant upregulation of MT1-MMP expression (at both gene and protein levels).

After treatment with collagen and insulin, cells overexpressing DDR2 demonstrated both increased DDR2 p-Tyr740 and total tyrosine phosphorylation (Malcor et al., 2018). In osteoblasts DDR2 can activate the transcription factor Runx2, via the p38 MAPK signaling pathway, regulating differentiation (Zhang et al., 2015). Type I collagen activated DDR2 increases the stability of the EMT driving factor SNAIL1, promoting invasion and migration of breast cancer cells *in vitro* and metastasis *in vivo* (Zhang et al., 2013). Furthermore, in triple negative breast cancer, H-Ras promotes EMT by downregulation of DDR1 expression via its transcriptional repressor of ZEB1 (Koh et al., 2015). In thyroid papillary cancer cells DDR2 activates ERK, increasing the stability of the EMT driving factor SNAIL1, reducing invasion and distant metastasis (Liang et al., 2017).

### Collagen-Independent Discoidin Domain Receptor Tyrosine Kinase 1 and Discoidin Domain Receptor Tyrosine Kinase 2 Signaling

The DDRs do in fact have activity independent of collagen-binding and receptor kinase activity, which can be stimulated by integrin, TGF-β, and insulin receptors. This independent activation can alter both cell adhesion, and differentiation (Miller et al., 2017; Vella et al., 2019). Insulin receptor (IR-A) and insulin-like growth factor (IGF-I) can bind directly with DDRs, with IGF-I-DDR heterodimer activity independent of collagen binding. Activation of insulin-like growth factor/insulin-like growth factor receptor (IGF-I/IGF-IR) leads to up-regulation of G-protein estrogen receptor (GPER) and DDR1 expression (Avino et al., 2016). IGF-1R, IGF-1, and insulin-like growth factor-2 (IGF-2) signaling through the PI3K/AKT/miR-199a-5p pathway up-regulates DDR1 (Belfiore et al., 2018). DDR1 interacts with the Par3/Par6 cell polarity complex through the carboxyl terminal PDZ binding motif, and thus controls actin activity between cells (Hidalgo-Carcedo et al., 2011). While it has been shown that DDR1 knockout reduces insulin receptor expression (inhibiting proliferation and migration) (Vella et al., 2017), the inverse is also true in that an increase in DDR1 protein expression can be induced through IGF-I overexpression (Matà et al., 2016).

### Discoidin Domain Receptor Tyrosine Kinase Dysregulation in Cancer

**FIGURE 8:**
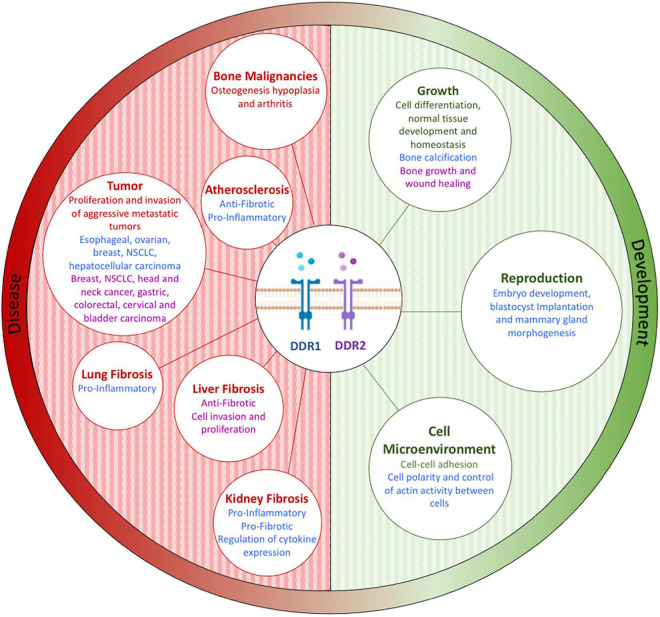
Discoidin domain receptor tyrosine kinase expression and/or activation plays a role in both physiological (e.g., development) and pathological (e.g., cancer, inflammation, and fibrosis) conditions by controlling key cellular processes, including protease production, cytokine secretion, cell migration, immune cell recruitment, and matrix production. Figure adapted and modified from Borza and Pozzi (2014).

Dysregulation of DDR1 and DDR2 has been observed in many solid tumor types (including breast, ovarian, liver, gastric, colorectal, lung, brain, cervical, hematological, head and neck, melanoma, bladder, kidney, and prostate), and can be associated with aggressive metastatic tumors (including breast and ovarian) and a poor prognosis (Gadiya and Chakraborty, 2018; Yeh et al., 2019; Lafitte et al., 2020; Majo and Auguste, 2021). As discussed above, DDR dysregulation leads to many alterations which influence tumor development, pathology, and key clinical characteristics (key altered clinicopathological features being treatment response and prognosis) (Borza and Pozzi, 2014; Mehta et al., 2021). DDR1 and DDR2 expression and dysregulation, in both physiological (e.g., development) and pathological conditions (including cancer, inflammation, and fibrosis), is summarized and highlighted in Figure 8 (Borza and Pozzi, 2014). In the following sections we summarize some of the key breast and ovarian cancer focused research characterizing DDR1 and DDR2 dysregulation.

### Discoidin Domain Receptor Tyrosine Kinase 1 and Discoidin Domain Receptor Tyrosine Kinase 2 in Breast Cancer

It has been shown that higher DDR1 protein expression is associated with breast cancer, promoting proliferation by suppressing antitumor immunity (Zhong et al., 2019). In the triple-negative breast cancer subtype (TNBC, or basal breast cancer) dysregulation of both DDR1 and DDR2 has been associated with increased invasion, and a poorer prognosis (Toy et al., 2015). In TNBC, collagen IV activated DDR1 induces increased cell surface expression of CD9, and secretion of metalloproteinases MMP-2 and MMP-9 which promote migration through the ECM (Castro-Sanchez et al., 2010, 2011). Also in TNBC, co-expression of DDR1 and Protein phosphatase 1 regulatory subunit 1B (PPP1R1B, also known as DARPP-32) inhibits tumor cell migration (Hansen et al., 2006). Inhibition of DDR1 expression can significantly enhance the chemosensitivity of breast cancer cells to genotoxic treatments (Saby et al., 2019). Interestingly, DDR1 signaling has also been shown to enhance chemoresistance of breast cancer cells via NFκB-mediated expression of cyclooxygenase-2 (COX-2), and downregulation of DDR1 significantly enhanced drug sensitivity (Das et al., 2006).

Looking at associations with clinical breast cancer parameters, DDR1 protein expression was not significantly associated with either disease-free survival (DFS), or overall survival (OS) (Ren et al., 2013). However, in postmenopausal breast cancer patients, the specific DDR1 kinase domain mutation R776W does correlate closely with a poor prognosis (Griffith et al., 2018). The H-Ras pathway can cause mesenchymal-like phenotype changes in breast epithelial cells, and H-Ras inhibits DDR1 expression through ZEB1, a transcriptional inhibitor of DDR1. This H-Ras/ZEB1/DDR1 network interacts to promote tumor progression (Koh et al., 2015).

Discoidin domain receptor Tyrosine Kinase 2 expression in breast cancer was found to be six times higher than in normal breast tissue, and importantly was a significant independent predictor of both recurrence and prognosis (Ren et al., 2013). Investigating DDR2 signaling pathways, the collagen-dependent protease pappalysin-1 (PAPP-1) plays a critical role in postpartum breast cancer, with increased IGF signaling resulting from PAPP-1-mediated degradation of IGFBP-4 and IGFBP-5, promoting DDR2 signaling (Slocum and Germain, 2019). Pregnancy-associated plasma protein-A (PAPP-A), overexpressed in more than 70% of breast cancers, activates DDR2 converting postpartum anti-proliferative collagen into tumor-promoting collagen (Slocum et al., 2019). DDR2 gene deletion in a breast cancer mouse models has been shown to increase anti-PD-1 therapy sensitivity, and the combination of anti-PD-1 and DDR2 tyrosinase inhibitor Dasatinib reduces tumor burden (Tu et al., 2019). Another inhibitor WRG-28 regulates DDR2, targeting the RTK extracellular domain, inhibiting the invasion of breast cancer cell migration (Grither and Longmore, 2018). Heat shock protein 47 (HSP47) can promote collagen maturation and deposition, and HSP47 expression in breast cancer cells enhances their invasive ability. Furthermore, HSP47 silencing can reduce the stability of DDR2, and inhibit the migration and invasion of (breast) cancer cells (Chen J. et al., 2019). Breast Cancer-Associated Mesenchymal stem/multipotent stromal Cells (BC-MSC) can promote metastasis by increasing collagen deposition, with DDR2 up-regulation reported in MSC’s and in metastatic cancers (Gonzalez et al., 2017). In metastatic bone marrow mesenchymal hepatocytes DDR2 maintains the fibroblast phenotype, promotes collagen deposition, which enhances cell migration and metastatic capacity (Gonzalez et al., 2017). In stromal Cancer-Associated Fibroblasts (CAFs), DDR2 promotes ECM and collagen fibrous tissue deposition, and enhances tumor cell invasion and metastasis (Corsa et al., 2016). Beclin-1 (a critical regulator of autophagosome formation) regulates DDR2 expression, reducing both DDR2 expression and pro-inflammatory mediator IL-1β in breast cancer (Morikawa et al., 2015). It has been shown in breast cancer that DDR2 expression is associated with hypoxia marker HIF-1α expression, with DDR2 expression and phosphorylation increased under intra-tumoral hypoxic conditions (Ren et al., 2014). These findings implicate DDR2 in the development of hypoxia-induced breast cancer, and metastatic development.

### Discoidin Domain Receptor Tyrosine Kinase 1 and Discoidin Domain Receptor Tyrosine Kinase 2 in Ovarian Cancer

It has been shown that DDR1 (protein) is highly expressed in serous ovarian cancers compared to normal ovarian epidermal tissues, with DDR1 mainly expressed in epithelial ovarian cancer (EOC) cells (Chung et al., 2017). In EOC, the protein expression of DDR1, Claudin-3 (CLDN3) and epithelial cell adhesion molecule (Ep-CAM) are all significantly up-regulated, suggesting that this upregulation is an early driver event of EOC (Heinzelmann-Schwarz et al., 2004). Furthermore, it has been shown that DDR1 over-expression is closely related to patients’ disease-free survival (DFS), with significantly higher DDR1 protein expression observed in high-grade and advanced tumors (Quan et al., 2011). In ovarian cancer tissues, expression of DDR1 is negatively correlated with the expression of miR-199a-3p, where miR-199a-3p inhibits DDR1 overexpression, drastically reducing the migration, invasiveness, and tumorigenicity of ovarian cancer cells (Deng et al., 2017).

Discoidin domain receptor Tyrosine Kinase 2 is highly expressed in ovarian cancer tissues and has been shown to enhance the invasive ability of tumor cells (Zhao et al., 2011). DDR2 upregulation was detected in 103 ovarian cancer tissues, correlates with tumor stage and peritoneal metastasis, and is an independent prognostic factor (Fan et al., 2016). Immunohistochemical detection of DDR2 in high-grade serous ovarian cancer (HGSOC) found DDR2 up-regulation in 14.10% (11 of 78) of cases (Ramalho et al., 2019). Investigating DDR2 signaling, collagen XIα1 is an ECM small fibrillar collagen regularly overexpressed in ovarian cancer (including cisplatin resistance or recurrent ovarian cancer) (Quan et al., 2011). Collagen XIα1 binding to both integrin α1β1 and DDR2 mediates chemoresistance by activating signaling that inhibits cisplatin-induced apoptosis in ovarian cancer cells (Rada et al., 2018). DDR2 expression can be induced by EMT factor Twist 1, promoting metastasis (Grither et al., 2018). Further supporting a role for DDR2 in tumor development, knockdown of the tumor invasion regulator N-Myc Downstream Regulated 1 (NDRG1) enhanced tumor cell adhesion, migration and invasion activities (without affecting cell proliferation) and significantly increasing DDR2 expression (in ovarian and cervical cell lines) (Zhao et al., 2011).

## Clinical Developments of Discoidin Domain Receptor Tyrosine Kinase 1 and Discoidin Domain Receptor Tyrosine Kinase 2 Targeted Therapy in Cancer

Interestingly, many DDR signaling pathways (including IGF-DDR) can be used to treat chronic inflammatory diseases, including cancer (Vella et al., 2019). While we have focused here on DDR1 and DDR2 in breast and ovarian cancer, they are increasingly important and relevant anti-cancer targets for multiple tumor types (Lafitte et al., 2020; Bhanumathy et al., 2021; Gao et al., 2021). Previously Merestinib (LY2801653; inhibiting DDR1/2 and MET, MST1R, FLT3, AXL, MERTK, TEK, ROS1, and MKNK1/2) has shown potent anti-tumor activity in clinical trials against multiple advanced cancers (Yan et al., 2013). However, there is only a single trial (NCT03292536) directly evaluating the anti-tumor activity of Merestinib against metastatic breast cancer (and none against ovarian cancer) (Table 1). Additionally, currently only one other trial has included HR + HER2- Breast Cancers in its study design (NCT02791334).

**TABLE 1:** Clinical trials of Merestinib targeting breast cancer.

No	Treatment ways	Target	NCT number	Title	Status	Conditions	Interventions	Phase	Study design	Enrollment	Study Start
1	Monotherapy	MET kinase inhibitor, anti-DDR1/2	NCT032 92536	Merestinib on bone metastases in subjects with breast cancer	Terminated	Bone metastases; breast cancer	Drug: Merestinib	Phase 1	(1) Allocation: N/A; (2) Intervention model: Single group assignment; (3) Masking: None (open label); (4) Primary purpose: Treatment	2	January 11, 2018
2	Combination therapy	MET kinase inhibitor, anti-DDR1/2	NCT027 91334	A study of anti-PD-L1 checkpoint antibody (LY3300054) alone and in combination in participants with advanced refractory solid tumors	Active, not recruiting	Solid tumor; microsatellite instability-high (MSI-H) solid tumor; cutaneous melanoma; pancreatic cancer; breast cancer (HR +HER2-)	Drug: LY3300054; drug: Ramucirumab; drug: Abemaciclib; drug: Merestinib; drug: LY3321367	Phase 1	(1) Allocation: Non-Randomized; (2) Intervention model: Parallel assignment; (3) Masking: None (open label); (4) Primary purpose: Treatment	(1) Allocation: Non-Randomized; (2) Intervention model: Parallel assignment; (3) Masking: None (open label); (4) Primary purpose: Treatment	June 29, 2016

## Conclusion and Perspectives

The current body of fundamental research demonstrating the high expression of DDR1 and DDR2 in multiple female tumors suggests that DDRs play a significant role in tumorigenesis and regulate the occurrence and development of breast and ovarian cancers, influencing chemotherapeutic resistance and survival of tumor cells, and mediate cell invasion and metastases. As highlighted by work in other tumour types, the use of DDR1 and DDR2 targeting compounds holds significant promise for targeted anti-cancer treatment, as either mono or combinational therapeutics. Further fundamental research exploring DDR1 and DDR2 in breast and ovarian cancer is needed, to expand our knowledge of mechanisms driving progression in these cancers. It is anticipated that additional DDR1/2 targeted clinical trials will further strengthen the clinical case for the use of targeted DDR anti-cancer therapeutics, to improve outcomes for patients with either breast cancer or ovarian cancer.

## Author Contributions

LC and XK: writing and editing. YF and SP: data curation. XW: investigation. EB and JB: methodology, writing, and editing. XL and JW: resources, funding acquisition, and project administration. All authors contributed to the article and approved the submitted version.

## Conflict of Interest

The authors declare that the research was conducted in the absence of any commercial or financial relationships that could be construed as a potential conflict of interest.

## Publisher’s Note

All claims expressed in this article are solely those of the authors and do not necessarily represent those of their affiliated organizations, or those of the publisher, the editors and the reviewers. Any product that may be evaluated in this article, or claim that may be made by its manufacturer, is not guaranteed or endorsed by the publisher.
